# Infective Endocarditis: Controversies and Convictions in the Surgical Treatment

**DOI:** 10.21470/1678-9741-2023-0014

**Published:** 2023-08-07

**Authors:** Kyriakos Spiliopoulos, Dimitrios Magouliotis, Andrew Xanthopoulos, Nikolaos S. Salemis

**Affiliations:** 1 Department of Cardiothoracic Surgery, University of Thessaly, Larissa, Greece; 2 Department of Perfusion Technology, Manipal College of Health Professions, Manipal, Karnataka, India; 3 Department of Cardiology, University of Thessaly, Larissa, Greece; 4 Breast Cancer Surgery Unit, Army General Hospital, Athens, Greece

Infective endocarditis (IE) is characterized by increased morbidity and mortality rates. The role of surgery as an adjunct to medical therapy depends on various factors including, among others, the virulence of the causative pathogen, the damage of surrounding tissues, and whether the infected valve is native or prosthetic. Although surgical treatment has been proposed in the early era of open heart surgery, the decision to operate is often difficult. Despite advances made over the years in prompt diagnosis, better antimicrobial therapy, intensive care management, and operative techniques, surgery remains challenging, accompanied by high complication rates. IE patients are usually severely ill, hemodynamically compromised due to the involvement of various organ systems.

In this context, we read with great interest the article recently published in the journal entitled “Early Mortality Predictors in İnfective Endocarditis Patients: A Single-Center Surgical Experience”, by Üstünışık ÇT et al.^[1]^, which presented an early mortality analysis in 122 surgically treated IE cases. The issue is very relevant, and we would like to take the chance to add some comments about the surgical management of this entity.

One of the main study findings was that in-hospital mortality was related with the presence of periannular abscess. The incidence of paravalvular abscess due to active IE increased in recent years. In order to prevent severe valvular annular destruction, it is essential to establish early the diagnosis of periannular abscess, often made by transesophageal echocardiogram. However, if the abscesses are of small size and located on the anterior aortic wall and mitral annulus, their diagnosis remains difficult. According to our experience, the presence per se of a paravalvular abscess is not a predictor of adverse early surgical outcome^[2]^. In general, operative outcomes depend on the ability of the surgeon to recognize and remove all infected tissues, and it seems that the development of low cardiac output syndrome (LCOS) has major influence on early mortality^[2,3]^. The abscess impact is determined by its size, location, and extent of the damaged surrounding tissues, all of which may cause LCOS. Difficulties and challenges of the surgical procedures are due to the radical exclusion of the abscess cavity from the circulation and the secure fixation of the implanted prosthesis on a friable tissue. Thus, IE caused by microorganisms prone to form abscesses, such as *Staphylococcus aureus* (SA) or coagulase-negative *Staphylococcus,* are best treated with early surgery. Additionally, in SA infection, the pathogen causes, beside a severe valvular damage, large vegetations and embolic complications. In this context, the question arises whether it’s reasonable in SA-infection to consider a vegetation-size < 10 mm as an indication for intervention, the up to now recommended size-limit by the guidelines. Nevertheless, the vegetation diameter as a sole indication for surgery in the absence of complications of heart failure or uncontrolled infection, aiming to prevent embolism, is controversially discussed. It has been shown that the interobserver variability of estimating the vegetation size is too high to guide the decision of performing surgery^[4]^. Apart from the maximal diameter, there are several other morphologic vegetation characteristics like its attachment width to the endocardial surface, mobility, shape, and consistency defined by echodensity, which affect the embolism incidence. Furthermore, the vegetation location (mitral located more prone to embolism than aortic), as well particular causative microorganisms (SA as mentioned before) have been found to be associated to embolic episodes^[4]^.

Regarding the optimal timing of surgery in patients with IE, the issue remains under debate. For the cardiac surgeon, the dilemma exists whether to proceed to early surgery in order to prevent the risk of emboli and severe heart failure or to delay surgical intervention until the state of infection is controlled and the risk of operation is accordingly reduced. Furthermore, in the everyday practice, lots of patients are referred for surgery from peripheral hospitals or general practitioners, after receiving there a wide spectrum of empiric antibiotic therapy prior establishing the definite diagnosis, and thus the exact timing of surgery is decided at the discretion of the surgeon depending mainly on the personal expertise and experience^[5]^.

Last but not least, due to the expansion of transcatheter aortic valve implantation (TAVI) procedures in lower-risk patients, TAVI-IE, although for the time being an infrequent complication, is expected to gain in importance. Fortunately, the assumption that TAVI patients are more prone to prosthetic valve endocarditis (PVE) due to their clinical risk profile like advanced age and comorbidities, technical issues of the procedure (transfemoral access, insertion of several catheters, lines, pacemaker leads), and the device itself (crimping, big valve-stent, post-procedural aortic regurgitation, higher rate of permanent pacemaker implantation) has not been confirmed, and the incidence rate of TAVI-IE appears to be similar to this of the conventional surgical PVE. However, surgical treatment of those IE patients poses new challenges for the surgeon^[6]^.

Conclusively, decision-making regarding indication and timing of surgical treatment of IE patients is often difficult and usually highly dependent on the expertise of the surgical team. Data from clinical studies dealing with the issue are limited due to the small patient populations and their retrospective nature. Ideal for definitive conclusions on this topic is an adequate high-quality prospective assessment supported by a sophisticated propensity scoring model evaluating comparable groups.

Brazilian Journal of Cardiovascular Surgery
